# Okinawa-Based Nordic Diet Decreases Plasma Levels of IAPP and IgA against IAPP Oligomers in Type 2 Diabetes Patients

**DOI:** 10.3390/ijms25147665

**Published:** 2024-07-12

**Authors:** Dovilė Pocevičiūtė, Bodil Roth, Bodil Ohlsson, Malin Wennström

**Affiliations:** 1 Cognitive Disorder Research Unit, Department of Clinical Sciences Malmö, Lund University, 214 28 Malmö, Sweden; dovile.poceviciute@med.lu.se; 2 Department of Internal Medicine, Skåne University Hospital, 205 02 Malmö, Sweden; bodil.roth@med.lu.se (B.R.); bodil.ohlsson@med.lu.se (B.O.); 3 Department of Clinical Sciences Malmö, Lund University, 214 28 Malmö, Sweden

**Keywords:** prevention, amylin, diet, IgA, T2D

## Abstract

Pancreas-derived islet amyloid polypeptide (IAPP) aggregates and deposits in the pancreas and periphery of Type 2 Diabetes (T2D) patients, contributing to diabetic complications. The excess IAPP can be removed by autoantibodies, and increased levels of immunoglobulin (Ig) G against IAPP have been reported in T2D patients. However, whether other Ig classes are also affected and if the levels can be managed is less known. This pre–post study examines IgA levels against IAPP oligomers (IAPP_O_-IgA) in T2D patients and assesses the impact of the Okinawa-based Nordic (O-BN) diet—a low-carbohydrate, high-fiber diet—on these levels after following the diet for 3 months. IAPP, IAPP_O_-IgA, and total IgA levels were measured in plasma and fecal samples from *n* = 30 T2D patients collected at baseline, after 3 months of diet, and after additional 4 months of unrestricted diets (a clinical follow-up). The IAPP and IAPP_O_-IgA levels were significantly lower after 3 months, with the latter also being significantly reduced at the clinical follow-up. The reduction in plasma IAPP and IAPP_O_-IgA levels correlated with reductions in plasma levels of metabolic and inflammatory markers. Hence, following the O-BN diet for at least 3 months is sufficient to reduce circulating IAPP and IAPP_O_-IgA levels, which may be principal in managing T2D.

## 1. Introduction

Type 2 Diabetes (T2D) is a chronic metabolic disorder characterized by persistent hyperglycemia and insulin resistance. The insulin resistance leads to a compensatory increase in insulin secretion, which in the long run exhausts the pancreatic beta cells, ultimately leading to the beta cell failure [1]. This failure in combination with the hyperglycemia and other metabolic alterations leads to microvascular (e.g., retinopathy, nephropathy, neuropathy) and macrovascular complications, with subsequent increased risk in cardiovascular diseases [2] and dementia [3]. One of the most effective measures in managing T2D is to alter the diet and reduce body weight. The typical Western diet consisting of high consumption of carbohydrates, saturated fat, and processed food promotes a lower gut microbial diversity, increases intestinal permeability, and induces metabolic and inflammatory impairments that are associated with several age-related disorders including the most common form of dementia—Alzheimer’s disease (AD) [4]. The replacement of a high-fat diet (HFD) with a standard diet for 2 months and 4 months led to weight loss and an improved or completely normalized, respectively, metabolic profile of T2D mice [5]. Similarly, the Okinawa-based Nordic (O-BN) diet, which emphasizes eating plant-based foods, has been shown to increase satiety and improve gastrointestinal (GI) symptoms and health-related quality of life as well as reduce body mass index and levels of metabolic (e.g., glucose) and inflammatory (e.g., interleukin (IL)-18) markers in T2D patients following the diet for 3 months [6]. Even a single breakfast based on the O-BN diet has been shown to increase satiety, improve glucose homeostasis, and lower levels of glucose-dependent insulinotropic polypeptide (GIP) in healthy volunteers [6].

The increased secretion of insulin in T2D patients also affects the secretion of a peptide called islet amyloid polypeptide (IAPP, or amylin), since it is co-secreted with insulin [7]. Under physiological conditions, the function of IAPP is to lower blood glucose levels following a meal, slowing down gastric emptying, inhibiting glucagon release, and inducing satiety through binding to its receptors in the area postrema in the brain [8]. However, when IAPP secretion increases, along with insulin secretion, it aggregates into higher structure soluble oligomers and insoluble fibrils. These IAPP oligomers have been associated with beta cell toxicity, apoptosis, and depletion, and depositions of insoluble aggregated IAPP have been identified in the pancreas of more than 90% of T2D patients [9]. Similar cytotoxic depositions have also been found in the kidneys and hearts of T2D patients, who also showed increased levels of circulating IAPP [10,11,12]. Moreover, a recent study has demonstrated IAPP oligomers in the skin of T2D patients and has provided evidence for an IAPP-driven peripheral neuropathy in a mouse model of the disease [13]. Interestingly, IAPP oligomers and plaques have also been identified in the brain capillaries and parenchyma of T2D patients and AD patients without apparent clinical diabetes [14]. The impact of IAPP on the brain, in particular the brain vasculature, has further been shown by us in studies demonstrating brain pericyte contraction and death in response to oligomeric IAPP uptake [15,16] and a strong correlation between the blood–brain barrier permeability and plasma IAPP levels in patients with AD [17].

The primary mechanism of oligomeric IAPP clearance in beta cells is through the autophagic pathway, but this process is impaired in T2D [18]. The amyloidogenic IAPP can also be neutralized and cleared by endogenous antibodies against the peptide, so-called autoantibodies. Indeed, several studies have described IAPP-autoantibodies in T2D patients; one group has reported significantly higher IAPP-autoantibody levels in T2D patients [19], while another group has identified autoantibodies which specifically recognized cytotoxic oligomers, but not monomers or fibrils, in T2D patients [20]. The role for autoantibodies in IAPP clearance was further demonstrated in vivo, showing that exogenous aggregated but not monomeric IAPP evoked autoantibody response in a mouse model of islet amyloidosis, which further prevented IAPP depositions as well as delayed onset of hyperglycemia and the induction of pro-inflammatory IL-1β [21]. To add, a recent study has reported an antibody generated from cultured human memory B cells that selectively clears oligomeric IAPP in murine T2D models, resulting in beta cell protection and improved glucose control [22]. We have, in our own studies, demonstrated that cases with AD pathology show increased plasma IAPP_O_-IgA levels, indicative of increased plasma oligomeric IAPP levels in these patients. We have also found a drastic reduction in IAPP_O_-IgA levels in a subset of the cases with AD pathology. These cases carried the high risk (for AD) gene *APOE4*, suggesting that these patients had a malfunctional clearance by IgA, leaving them exposed to higher levels of circulating cytotoxic IAPP oligomers [23]. Interestingly, we found no impact on IAPP_O_-IgM or IAPP_O_-IgG levels in either AD patients or *APOE4* carriers, suggesting a specific role for IgA in clearance of oligomeric IAPP. Since IgA is primarily found in the gut and IAPP is also (beside pancreas) produced in the GI tract [24], we hypothesized that dietary effects on the GI tract can influence IAPP secretion and IAPP_O_-IgA production. To test our hypothesis, we analyzed the levels of IAPP and IAPP_O_-IgA in the plasma and feces of T2D patients, a patient group characterized by IAPP pathology, before and after 3 months of the O-BN diet. We also investigated if the levels remain altered 4 months after ending the diet and if the IAPP and IAPP_O_-IgA levels in plasma and feces correlate with alterations in metabolic, inflammatory, and brain markers.

## 2. Results

### 2.1. Clinical Characteristics

This study included 30 patients (of whom 17 were women) with T2D, mean age 58 ± 8 years, mean disease duration 10 ± 8 years [6], and mean body mass index (BMI) 29.8 ± 4.2 kg/m^2^ at baseline (Table 1). In detail, 19 out of 30 patients had at least one diabetic complication, from which retinopathy was the most common (Table 1). Additionally, 10% of patients managed their T2D with diet alone, 13% with diet and insulin, 50% with diet and metformin, and 27% with diet and both insulin and metformin (Table 1).

### 2.2. The Okinawa-Based Nordic Diet Decreases Plasma Levels of IAPP

**Figure 1:**
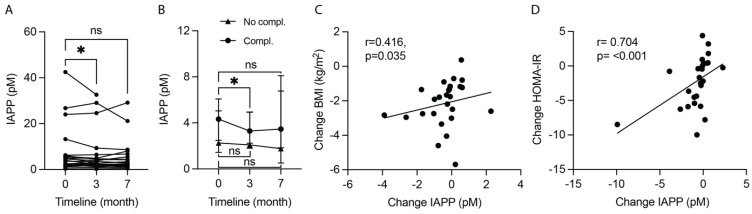
The intervention with the Okinawa-based Nordic (O-BN) diet for 3 months significantly reduces plasma islet amyloid polypeptide (IAPP) levels in Type 2 Diabetes (T2D) patients, and the reduction is associated with lowered body mass index (BMI) and improved insulin resistance (HOMA-IR). The graph (**A**) illustrates a significant reduction in plasma IAPP levels after following the O-BN diet for 3 months, but the levels return to baseline levels 4 months after the dietary intervention and unrestricted eating (7th month). Interestingly, this significant reduction in plasma IAPP levels after 3 months of dietary intervention is only seen in T2D patients with at least one diabetic complication, but not in patients with no complications (**B**). The reduction in plasma IAPP levels correlates significantly with reductions in BMI (**C**) and HOMA-IR (**D**). The data are shown as individual values (**A**,**C**,**D**) and summary data with median and 95% CI (**B**), and they were analyzed with Wilcoxon matched-pairs signed rank test (**A**,**B**) and Spearman’s correlation test (**C**,**D**). ns—not significant. * Significant at *p* ≤ 0.05 level.

The investigation was initiated by analyzing IAPP levels in plasma from T2D patients following the O-BN diet for 3 months, with a clinical follow-up after another 4 months of unrestricted diets (the *clinical follow-up*). The IAPP levels did not alter after 3 months (*p* = 0.159) or at the clinical follow-up (*p* = 0.378) compared to baseline. However, two patients showed extremely high levels (60- and 170-times higher plasma IAPP levels than the median at baseline) and were hence considered as outliers. After removal of the two outliers, there was a significant reduction in IAPP levels after 3 months (*p* = 0.027). The IAPP levels returned to baseline levels at the clinical follow-up (*p* = 0.189) (Figure 1A). No difference in IAPP levels was found between the different T2D management strategies (i.e., diet alone, diet and metformin, diet and insulin, or diet and combination of metformin and insulin) at baseline, after 3 months, or at the clinical follow-up (*p* = 0.810, *p* = 0.808, and *p* = 0.298, respectively). Interestingly, after stratifying the cohort into groups with and without diabetic complications, the diet-induced reduction in IAPP levels was seen only in the group with complications (*p* = 0.031) and not in the complication-free group (*p* = 0.646) (Figure 1B). Since the O-BN diet significantly reduces weight [6] and we noted a significant correlation between IAPP and BMI at baseline (Table 2), we also analyzed IAPP levels using ANCOVA. No significant difference was found when comparing IAPP levels between baseline, after 3 months, and at the clinical follow-up after correcting for age, gender, *APOE4* status, and BMI (*p* = 0.726), or after additionally correcting for “diabetic complications” (*p* = 0.646).

### 2.3. Plasma IAPP Levels Correlate with Metabolic and Inflammatory Markers

Further correlation analyses of values at baseline demonstrated correlations between IAPP levels and various metabolic and inflammatory plasma markers, in particular insulin, insulin resistance, CRP, and GGT (Table 2). After correcting for BMI, age, gender, and *APOE4* status (variables known to affect metabolism [25,26] and IgA levels [23,27]), none of the correlations remained significant except for IFNγ, while new correlations with mainly inflammatory plasma markers were revealed (Table 2). When we instead analyzed the change in IAPP concentration between baseline and after 3 months of diet (changes further on), we again noted a positive correlation with BMI (Figure 1C) as well as insulin and insulin resistance (Figure 1D), glucose, and several interleukins, but a negative correlation with NfL (Table 2). After correcting the correlations for BMI (age, gender, and *APOE4* status were considered as constant variables), only the correlation with IL2 remained significant (Table 2). We found no significant correlations between IAPP and metabolic markers CCK, ghrelin, glucagon, GLP-1, HDL, leptin, PYY, triglycerides, visfatin, short-chain fatty acids acetic acid, isobutyric acid, isovaleric acid, propionic acid, liver markers ALT and haptoglobin, peripheral inflammation markers calprotectin and PAI-1, and psychological well-being neither at baseline nor when analyzing changes in concentrations, before or after the corrections.

### 2.4. The Okinawa-Based Nordic Diet Decreases Plasma Levels of IgA against Oligomeric IAPP

**Figure 2:**
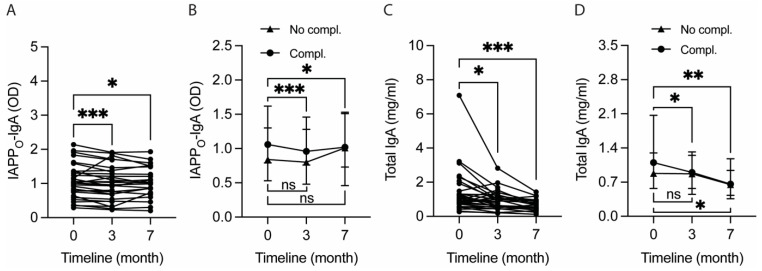
Intervention with the Okinawa-based Nordic (O-BN) diet for 3 months significantly reduces plasma total IgA levels and IgA levels against toxic islet amyloid polypeptide (IAPP) oligomers (IAPP_O_-IgA) in Type 2 Diabetes (T2D) patients, and the levels remain significantly reduced after 4 months post-diet intervention. The graphs (**A**,**C**) demonstrate significantly reduced plasma IAPP_O_-IgA and total IgA levels after following the O-BN diet for 3 months, with the levels remaining significantly reduced even after 4 months post-dietary intervention and unrestricted eating (7th month). The significant reductions in plasma IAPP_O_-IgA and total IgA levels at the end of the intervention and 4 months post-intervention (7th month) are foremost seen in patients with at least one diabetic complication (**B**,**D**). The data are shown as individual values (**A**,**C**) and summary data with median and 95% CI (**B**,**D**), and they were analyzed with Wilcoxon matched-pairs signed rank test. ns—not significant. * Significant at *p* ≤ 0.05 level. ** Significant at *p* ≤ 0.01 level. *** Significant at *p* ≤ 0.001 level.

Next, to investigate if the diet also reduced the levels of autoantibodies against IAPP, we investigated the plasma levels of IgA against IAPP monomers (IAPP_M_-IgA) and oligomers (IAPP_O_-IgA). The IAPP_M_-IgA levels did not alter after 3 months (*p* = 0.102) or at the clinical follow-up (*p* = 0.098). In contrast, the IAPP_O_-IgA levels were significantly reduced after 3 months (*p* < 0.001) and remained significantly reduced at the clinical follow-up (*p* = 0.045) (Figure 2A). Similarly to the plasma IAPP levels, the reduction in IAPP_O_-IgA levels both after 3 months and at the clinical follow-up was only seen in the group with diabetic complications (Figure 2B). Also, the total IgA levels were significantly reduced both after the 3-month-long diet (*p* = 0.012) and at the clinical follow-up (*p* < 0.001) (Figure 2C). Again, the reduction in total IgA levels both after 3 months (*p* = 0.013) and at the clinical follow-up (*p* = 0.003) was foremost seen in the group with diabetic complications, although the levels were also reduced in complication-free patients at the clinical follow-up (*p* = 0.012) (Figure 2D). Neither IAPP_M_-IgA nor IAPP_O_-IgA levels differed between T2D management strategy groups at baseline (*p* = 0.854 and *p* = 0.287, respectively), after 3 months (*p* = 0.802 and *p* = 0.341, respectively), or at the clinical follow-up (*p* = 0.395 and *p* = 0.690, respectively).

### 2.5. Levels of IAPP Autoantibodies Correlate with Metabolic and Inflammatory Markers

Subsequent analyses also revealed correlations between the levels of IAPP-autoantibodies and T2D-associated plasma biomarkers (Table 3). The IAPP_M_-IgA levels correlated positively with glucose, total IgA, and the liver damage markers GGT and ALT at baseline, and the change in IAPP_M_-IgA levels correlated positively with changes in the levels of IAPP, glucagon, and GIP (Table 3). Similarly, the IAPP_O_-IgA levels correlated positively with the metabolic markers glucose, glucagon, and GIP, as well as with inflammatory total IgA, CRP, and GGT (Table 3), and the change in IAPP_O_-IgA levels correlated positively with the change in insulin resistance (Table 3). Neither IAPP_M_-IgA nor IAPP_O_-IgA levels correlated significantly with BMI at baseline (r = 0.360, *p* = 0.051 and r = 0.305, *p* = 0.102, respectively) or in terms of changes in concentrations (r = 0.117, *p* = 0.553 and r = 0.107, *p* = 0.587, respectively). Of note, total IgA levels also did not correlate significantly with BMI at baseline (r = 0.168, *p* = 0.374) or in terms of changes (r = 0.163, *p* = 0.407). When the baseline correlations were adjusted for age, gender, *APOE4* status, and BMI, most of the correlations with metabolic markers were lost for both IAPP_M_-IgA and IAPP_O_-IgA, but significant correlations between the IAPP_M_-IgA and inflammation markers and between the IAPP_O_-IgA and NfL levels were detected instead (Table 3). When changes in concentrations were analyzed, IAPP_M_-IgA levels correlated positively with IAPP levels and both IAPP_M_-IgA and IAPP_O_-IgA correlated with various metabolic plasma markers (Table 3). Only the correlation between IAPP_M_-IgA and triglycerides remained after BMI correction, while a correlation with insulin resistance was detected instead. Similarly, the correlations of IAPP_O_-IgA levels with metabolic markers were lost after BMI correction, and instead a correlation with isobutyric acid was found (Table 3). We found no significant correlations between IAPP_M_-IgA or IAPP_O_-IgA and metabolic markers C-peptide, CCK, cholesterol, ghrelin, GLP-1, HDL, leptin, LDL, PYY, resistin, visfatin, short-chain fatty acids acetic acid, butyric acid, isovaleric acid, propionic acid, liver marker haptoglobin, peripheral inflammation markers calprotectin, IL4, IL12p70, PAI-1, and psychological well-being, neither at baseline nor when analyzing changes in concentrations, before or after the corrections.

### 2.6. Reduction in Plasma IAPP_O_-IgA Levels after 3 Months Is Selectively Seen in Non-APOE4 Carriers

**Figure 3:**
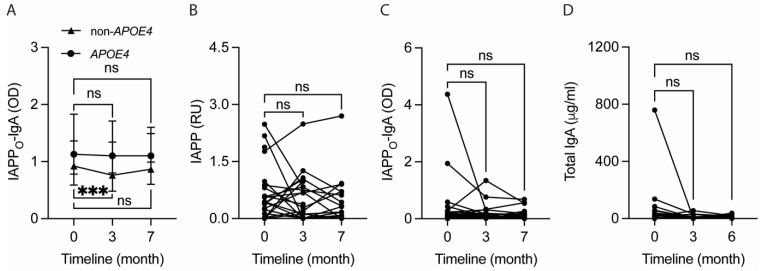
Intervention with the Okinawa-based Nordic (O-BN) diet for 3 months significantly reduces plasma IgA levels against toxic islet amyloid polypeptide (IAPP) oligomers (IAPP_O_-IgA) in Type 2 Diabetes (T2D) patients not carrying the apolipoprotein E 4 (*APOE4*) gene, but no effect is found on fecal levels of IAPP, IAPP_O_-IgA, and total IgA in all T2D patients. The graph (**A**) demonstrates that the significant reduction in plasma IAPP_O_-IgA levels after 3 months of the O-BN diet is only found in T2D patients who do not carry a high-risk gene, *APOE4*, for cardiovascular and Alzheimer’s diseases. The analyses of fecal samples show that 3 months of the O-BN diet has no profound effect on levels of IAPP (**B**), IAPP_O_-IgA (**C**), or total IgA (**D**). The data are shown as summary data with median and 95% CI (**A**) and as individual values (**B**–**D**), and they were analyzed with Wilcoxon matched-pairs signed rank test. ns—not significant. *** Significant at *p* ≤ 0.001 level.

Since we have previously reported an effect of the *APOE* genotype on IAPP-autoantibody levels in AD patients, we also investigated if plasma IAPP_O_-IgA levels in T2D patients are dependent on *APOE4* status. The IAPP_O_-IgA levels did not differ between *APOE4* carriers (*n* = 11) and non-*APOE4* carriers (*n* = 19) at baseline (*p* = 0.328), after 3 months (*p* = 0.200), or at the clinical follow-up (*p* = 0.154). However, while the IAPP_O_-IgA levels were unaltered in *APOE4* carriers after 3 months (*p* = 0.213) and at the clinical follow-up (*p* = 0.237), we noted significantly lower IAPP_O_-IgA levels in non-*APOE4* carriers after 3 months (*p* < 0.001) and a trend towards significantly lower levels at the clinical follow-up (*p* = 0.088) (Figure 3A).

The plasma IAPP_O_-IgA levels in non-*APOE4* carriers correlated positively with plasma levels of IAPP (r = 0.490, *p* = 0.039), CRP (r = 0.659, *p* = 0.002), GGT (r = 0.659, *p* = 0.002), glucose (r = 0.640, *p* = 0.003), GIP (r = 0.458, *p* = 0.049), C-peptide (r = 0.516, *p* = 0.024), insulin (r = 0.458, *p* = 0.049), and insulin resistance (r = 0.581, *p* = 0.009) at baseline. In contrast, the IAPP_O_-IgA levels also correlated with levels of NfL (r = 0.664, *p* = 0.026) and glucagon (r = 0.645, *p* = 0.032) at baseline, but only in *APOE4* carriers.

### 2.7. Fecal IAPP_O_-IgA Levels Correlate with Gut Inflammation Markers

Finally, we analyzed the impact of the diet on IAPP, IAPP_O_-IgA, total IgA, and albumin levels in feces, where the latter variable, i.e., albumin, is considered to be an indicator of GI leakage and inflammation [28]. Levels of IAPP (Figure 3B), IAPP_O_-IgA (Figure 3C), total IgA (Figure 3D), or albumin were not affected after 3 months (*p* = 0.940, *p* = 0.133, *p* = 0.136, and *p* = 0.350, respectively) or at the clinical follow-up (*p* = 0.356, *p* = 0.685, *p* = 0.346, and *p* = 0.709, respectively).

The fecal levels of IAPP_O_-IgA correlated positively with fecal total IgA (r = 0.891, *p* < 0.001), albumin (r = 0.422, *p* = 0.023), and calprotectin (r = 0.620, *p* < 0.001) levels at baseline. Also, the changes (3 vs. 0 months) in fecal IAPP_O_-IgA levels correlated positively with changes in total IgA (r = 0.656, *p* < 0.001) and albumin (r = 0.451, *p* = 0.018) levels. No correlation between the fecal levels of IAPP and IAPP_O_-IgA was detected at baseline (r = −0.084, *p* = 0.665) or when the change in levels after the 3 months was analyzed (r = −0.055, *p* = 0.790).

## 3. Discussion

The aim of this study was to investigate if fasting plasma levels of IAPP and autoantibodies against toxic IAPP oligomers in T2D patients alter in response to the O-BN diet. Indeed, we found a significant reduction in IAPP levels already after 3 months in this cohort, which coheres with the previously shown reduction in levels of other metabolic hormones (e.g., insulin, leptin) [6]. The reduction in both IAPP and insulin levels might be due to the low carbohydrate and high fiber content in the diet, considering that the co-secretion of these hormones increases upon elevated blood glucose levels [7]. The IAPP levels also correlated strongly with CRP levels at baseline and the reduction in IAPP levels correlated with the reduction in levels of several pro-inflammatory cytokines, suggesting a link between circulating IAPP levels and inflammation. The association between IAPP and CRP has been previously described in AD patients [23] and healthy individuals [29], but whether the inflammation is induced by elevated IAPP levels or vice versa is not known. However, it is well-established that inflammation induces insulin resistance [30], which leads to an increased co-secretion of insulin and IAPP. On the other hand, a substantial increase in IAPP secretion enhances IAPP aggregation, contributing to cell toxicity and thereby inflammation. Notably, the reduction in IAPP levels as well as in other metabolic and inflammatory markers might have occurred not due to the change in diet per se, but due to a significant reduction in weight. Obesity is characterized by low-grade inflammation, with adipose tissue releasing many inflammatory markers which in turn contribute to insulin resistance and other metabolic disturbances [31]. Thus, a reduction in BMI could in turn lower IAPP levels. Our results are in line with this thought, as we found a significant correlation between reduced IAPP levels and reduced BMI after 3 months; additionally, after correcting for BMI (along with age, gender, and *APOE4* status), the significant reduction in plasma IAPP levels after 3 months was lost. Hence, it seems that the O-BN diet reduces BMI, which in turn contributes to a reduction in IAPP levels.

In similarity to the reduced IAPP levels, the levels of IAPP_O_-IgA also decreased after 3 months of the O-BN diet. This result should be viewed from the perspective that changes in autoantibody levels, in general follow, changes in endogenous antigen levels [32]. To exemplify this, an islet amyloidosis mice model which was immunized with the N-terminal peptide of IAPP (the terminal being highly exposed in IAPP oligomers) increased the production of IgG against aggregated, but not monomeric IAPP [21], suggesting a direct association between IAPP_O_-autoantibody production and oligomeric IAPP levels. Unfortunately, plasma levels of IAPP oligomers are difficult to analyze with no reliable methods available, thus we cannot provide evidence for a linear relationship between IAPP_O_-IgA and oligomeric IAPP levels. Nevertheless, the IAPP_M_-IgA levels (i.e., IgA directed against IAPP monomers) after the 3-month diet correlated positively with the reduction in IAPP levels (possibly constituting of both IAPP monomers and oligomers), which supports the idea of a linear relationship between the levels of IAPP-IgA autoantibodies and their autoantigen IAPP.

The significance of the found reduction in IAPP and IAPP_O_-IgA levels after 3 months of the O-BN diet should further be viewed from the notion that abnormal IAPP secretion and circulation of IAPP oligomers in T2D patients is associated with IAPP deposition and cell toxicity, which potentially contribute to diabetic complications (such as neuropathy, retinopathy, and vascular complications) found in these patients. From this perspective, a reduction in oligomeric IAPP levels caused by dietary changes could reduce or even prevent such diabetic complications. Interestingly, when analyzing our cohort, we noticed that the diet-induced reduction in plasma levels of IAPP and IAPP_O_-IgA was foremost found in patients with one or more diabetic complications, suggesting a beneficial impact foremost for patients in more advanced stages of the disease. The patients with complications also showed higher (although not significantly higher) median levels of IAPP and IAPPo-IgA at baseline, which again point towards a link between IAPP pathology and diabetic complications. It should, however, be noted that the size of our cohort, especially when divided upon the presence of at least one diabetic complication, is rather small. We, therefore, do not exclude the possibility that the diet also reduces IAPP and IAPP_O_-IgA levels in patients without diabetic complications, albeit in a rather low-grade (nonetheless important) manner. It should further be noted that the lowered IAPP plasma levels after the diet reverted back once the patients stopped following the diet (detected at the follow-up study), which is in agreement with previous results demonstrating a reduction in metabolic markers only after 3 months and not at the clinical follow-up (reviewed in [6]). In addition, although IAPP_O_-IgA levels were still significantly lower at the follow-up compared to baseline, the magnitude of reduction was not as after 3 months. From this, we can conclude that the strong changes in both IAPP and IAPP_O_-autoantibody levels are indeed affected by the diet itself. We can also conclude that the effect of the diet on IAPP_O_-autoantibody levels persists longer but decreases with time, possibly due to a return of oligomeric IAPP.

The biological mechanisms behind the dietary effects of the O-BN diet are still to be determined. But since the diet contains a moderately low carbohydrate energy content with higher contents of fiber, fat, and protein, the content of high-glycemic-index food is reduced radically, and gluten content is decreased. The reduced sugar content may play an important part in the effect of the O-BN diet, as it is known that sugar-rich diets, especially rich in fructose-containing sugars, are associated with harmful effects, such as increased insulin resistance, development of metabolic syndrome, and altered microbiota composition [33]. It is further likely that the high intake of whole-grains in the O-BN diet is of importance, as high-fiber, whole-grain diets are, in general, more beneficial than other interventions, independent of carbohydrate intake. For example, the Mediterranean diet, which is (just like the O-BN diet) rich in whole-grain, non-processed, plant-derived foods and lower in red meat, meat products, sweets, salt, high-fat dairy, and refrained grains [34], has been shown to have a beneficial impact on T2D clinical parameters [35]. Of note, although the O-BN and the Mediterranean diets resemble each other, they differ in food composition as the diets derive from different regions (Japan and Nordic countries vs. the Mediterranean region). In addition, while the carbohydrate percentage is the same in the two diets, the protein content is higher and the fat content is lower in the O-BN diet (41). The O-BN diet is also rich in omega-3 fats, with a high monounsaturated-to-saturated fat ratio [6], which were shown to improve the levels of total cholesterol, triglycerides, HDL, HbA1c, and CRP in T2D patients [36]. The diet is, however, lower in isoflavone content compared to the original Okinawa diet [34]. These types of flavonoids are common in soy and soy products (e.g., tofu) and have been shown to exhibit strong anti-inflammatory effects and a lower risk of cardiovascular disease [34]. They have therefore been used in women transitioning from pre- to post-menopause to ameliorate emerging metabolic syndrome [37,38]. Hence, we speculate that a combination of the O-BN diet and other anti-inflammatory compounds, such as isoflavones, could provide a stronger effect in ameliorating metabolic syndrome. Interestingly, we have also recently shown that the diet induces changes in metabolomics and gut microbiota. These changes correlated with weight reduction, improved glucose and lipid homeostasis, and lowered IL-18 levels [39], and thereby highlighted previously reported tight associations between the gut microbiota and chronic inflammation, insulin resistance, and cardiovascular/T2D diseases [40]. Importantly, the diet-induced changes in our cohort, including changes in microbiome [39] as well as in hormones and inflammation markers [6], were reversed at the clinical follow-up, which supports the idea that gut health is, at least in part, involved in the biological mechanisms implicated in T2D.

In our previous study on AD patients, we demonstrated a strong significant reduction in IAPP_O_-IgA levels in *APOE44* carriers, while the reduced levels in *APOE34* carriers did not reach significance compared to *APOE33* carriers [23]. Unfortunately, none of the individuals in the current study were *APOE44* carriers, and hence we could not verify the previous *APOE44* results [23]. However, we noticed that the diet-induced reduction in IAPP_O_-IgA levels was exclusively seen in non-*APOE4* carriers. The absence of such a reduction in *APOE4* carriers might be due to binding and stabilization of IAPP oligomers by APOE4 [41], possibly affecting the recognition of oligomers by autoantibodies and further attenuating their clearance. Interestingly, previous studies have shown that *APOE4* is associated with an increased risk of cardiovascular diseases [42], poorer outcomes after disease or injury in the peripheral nervous system [43], as well as the frequency and severity of diabetic neuropathy [44,45] in patients with diabetes. Given the suggested role for oligomeric IAPP in diabetic complications such as neuropathy [13], it is tempting to speculate that the attenuated IAPP clearance seen in *APOE4* carriers may be implicated in this link between *APOE4* and diabetic complications.

Finally, both IAPP and IAPP_O_-IgA were detected in fecal samples, which supports the idea that IAPP is produced in the GI tract [24]. However, although we noted a reduction in the levels of both after 3 months of dietary intervention, none of them were significantly lowered, suggesting that the diet does not strongly affect the IAPP production in the GI tract. The diet also did not alter albumin levels in feces. Albumin under normal conditions is restricted from entering the GI lumen through the capillary endothelium. However, increased capillary permeability due to, e.g., inflammation, increases the leakage of albumin into the GI tract [28]. It is therefore interesting that we noted a correlation between albumin and IAPP_O_-IgA levels in feces, both when analyzing the baseline values and the change in levels after the 3-month diet, as it may implicate that oligomeric IAPP toxicity plays a role in gut leakage. Notably, we found no correlation between plasma and fecal levels of IAPP or IAPP_O_-IgA, which suggests that the productions of IAPP and IAPP-IgA autoantibodies in the GI tract are separate events from pancreatic IAPP production and systemic clearance.

## 4. Limitations

The results found in our study are based on a rather limited number of patients, and further studies on larger cohorts are needed to verify our findings. For example, to investigate the potential influence of medications (such as metformin and insulin treatments) on diet outcome, the groups need to be significantly larger. It should further be pointed out that the protocol of feces preparation included many methodological steps (homogenization, separation, and filtration) and the consistence of feces varied. Hence, although we normalized the values against measured protein concentrations, we cannot entirely exclude the possibility that the many methodological steps may have affected the results.

## 5. Materials and Methods

### 5.1. Individuals Included in the Study

This current study was performed on fasting plasma and fecal samples from a patient cohort which has already been described in previous studies [6,39,46,47,48]. The cohort consisted of 30 (17 women) patients with T2D from southern Sweden. The patients were delivered the same diet for 3 months (nutrition composition is provided in Table 4), except breakfast, which they had to prepare themselves (nutrition composition is provided in Supplementary Table S1). The patients were allowed to eat three meals a day and two snacks, consisting of a variety of fruits, berries, and seeds. The meal content has previously been described in more detail [6] (Supplementary Table S2).

The daily average calorie content of the meals (kcal) and the energy percentage (E%) are given for nutrients based on the intake of a normal week. Recommendations for a traditional diet according to the Nordic Nutrition Recommendations are shown for comparison (http://dx.doi.org/10.6027/Nord2014-002. accessed on 5 July 2024). Recommendations for total daily energy intake are not given since they are individual and differ between subjects.

Plasma and fecal samples were collected from 30 patients at baseline, i.e., the start-point of the study (0 month), 30 patients after 3 months of eating the diet (3rd month), and 23 patients after another 4 months of unrestricted diets (7th month) (called further on the clinical follow-up). The plasma levels of metabolic markers (C-peptide, cholecystokinin (CCK), cholesterol, ghrelin, glucagon, glucagon-like peptide-1 (GLP-1), glucose, glucose-dependent insulinotropic polypeptide (GIP), hemoglobin A1C (HbA1c), high-density lipoprotein (HDL), insulin, leptin, low-density lipoprotein (LDL), polypeptide YY (PYY), resistin, triglycerides, and visfatin), short-chain fatty acids (acetic, butyric, isobutyric, isovaleric, and propionic acids), liver markers (alanine aminotransferase (ALT), gamma-glutamyl transpeptidase (GGT), and haptoglobin), peripheral inflammation markers (C-reactive protein (CRP), calprotectin, interferon γ (IFNγ), interleukins (IL1α, IL1β, IL2, IL4, IL12p70, IL18), plasminogen activator inhibitor-1 (PAI-1), and tumor necrosis factor α (TNFα)), brain changes (neurofilament light chain (NfL), psychological well-being), as well as fecal levels of calprotectin and zonulin in feces have been previously measured and described [6,39,46,47,48,49,50] (Supplementary Table S3).

### 5.2. APOE Genotyping

*APOE* genotyping was performed in collaboration with Henrietta Nielsen from Stockholm University and was based on a protocol described in our previous study [27]. Shortly, the DNA from plasma samples was extracted using the QIAamp DNA Blood Kit (Qiagen, Hilden, Germany) and the concentration and quality were assessed using a NanoDrop One/OneC Microvolume UV-Vis Spectrophotometer (Thermo Scientific, Madison, WI, USA). Following the DNA extraction, the rs429358 and rs7412 variants of the *APOE* gene were targeted with Taqman Universal polymerase chain reaction (PCR) Master Mix No AmpErase UNG (Applied Biosystems, Carlsbad, CA, USA) and TaqMan single nucleotide polymorphisms genotyping assays (Thermo Scientific, Carlsbad, CA, USA). The amplification was performed using the QuantStudio 5 Real-Time PCR System, 384-well format (Applied Biosystems, Singapore), and the results were analyzed using Thermo Fisher Cloud software (v1.4.3).

### 5.3. Stratification of the Cohort

The cohort was stratified upon *APOE4* carriers and non-*APOE4* carriers, patients with at least one diabetic complication (*n* = 19) versus complication-free (*n* = 11) patients, as well as upon their T2D management. The *APOE4* carriers included patients with ε24 (*n* = 3) and ε34 (*n* = 8) *APOE* alleles, while non-*APOE4* carriers carried ε23 (*n* = 5) and ε33 (*n* = 14) *APOE* alleles. The diabetic complications have been previously described [48] and included retinopathy, microalbuminuria, peripheral neuropathy, macroangiopathy, autonomic neuropathy, and/or GI dysmotility (Table 1).

### 5.4. Preparation of IAPP Monomers and Oligomers

The IAPP monomers were prepared by dissolving the lyophilized human IAPP_1–37_ peptide (AlexoTech, Umeå, Sweden) in dimethyl sulfoxide to a concentration of 2.5 mM, water-sonicating for 10 min, and further diluting with Dulbecco’s phosphate-buffered saline (DPBS) to a concentration of 100 µM. The IAPP oligomers were prepared by solubilizing the lyophilized human IAPP_1–37_ peptide in 20 mM NaOH (pH 12). The pH was adjusted to pH 7 by diluting the solution in a phosphate buffer to a concentration of 100 µM. Thereafter, the IAPP preparation was agitated for 20 min at RT, followed by centrifugation at 14,000× *g* for 10 min (Biofuge 13, Heraeus Sepatech, Osterode, Germany) at 4 °C. The lower fraction (50 µL) was collected and stored at −80 °C. This fraction was further used as IAPP oligomers. Before use in the experiments, the concentration of IAPP oligomers was determined using a Pierce BCA Protein Assay Kit (Thermo Scientific, Rockford, IL, USA). Both the monomeric and oligomeric IAPP preparations were evaluated by Western blot using an antibody cocktail comprised of rabbit anti-human IAPP primary antibodies (Peninsula Laboratories, T4149, San Carlos, CA, USA and Abcam, Ab254259, Fremont, CA, USA), each diluted 1:1000 in a blocking buffer (3% fish gelatine) (Supplementary Figure S1).

### 5.5. Preparation of Fecal Homogenates

Fecal homogenates were prepared according to a published protocol [51], with several modifications. In short, we defrosted each sample, mixed thoroughly, and weighed out approximately 1.2 g of the sample. To this, we added PBS containing protease inhibitors (Merck, Darmstadt, Germany) at a 2:1 ratio with the fecal sample as well as a mixture of stainless 1.4 mm and zirconium oxide 2.0 mm beads. The mixture was left to stand for 5 min to allow the fecal sample to soften and then the sample was thoroughly vortexed with beads. The mixture was then centrifuged at 15,000× *g* for 5 min, followed by transferring of the supernatant to a fresh tube. Afterwards, to remove smaller debris and bacteria, we added PBS at a 3:1 ratio with the supernatant and filtered it using a syringe filter of 0.45 µm pore size (Sarstedt, Helsingborg, Sweden). Finally, we collected the filtered samples and performed total protein concentration determination using the Pierce BCA Protein Assay Kit.

### 5.6. Detection of IAPP in Plasma and Fecal Samples

The plasma and fecal IAPP levels were determined using Human Amylin ELISA Kit (Merck, Darmstadt, Germany) according to the manufacturer’s instructions. Approximately one third of fecal samples were below the detection range; these missing values were instead replaced with the lowest detected value divided by 10. Also, due to high variability in fecal viscosity, the total protein content in fecal samples was normalized following the IAPP detection, and the fecal IAPP levels were expressed as relative units (RU).

### 5.7. Detection of IAPP-IgA in Plasma and Fecal Samples

The autoantibodies were detected by an indirect ELISA based on previously published protocols in IAPP-autoantibody studies, including our own [19,21,23]. In short, 96-well, optically clear, flat-bottom microplates (Thermo Scientific, Roskilde, Denmark) were coated with either IAPP monomers (IAPP_M_) or oligomers (IAPP_O_), diluted with PBS to a concentration of 1 mg/L, and incubated overnight at 4 °C. The plates were then rinsed three times with 0.05% Tween in PBS (PBST). Non-specific binding sites were blocked with a blocking buffer consisting of 1% bovine serum albumin (BSA, Merck, Kankakee, IL, USA) in PBST for 1 h at RT and thereafter rinsed three times with PBST. Then, plasma or fecal samples were applied and incubated for 2 h at RT with agitation. Plasma samples for IAPP_M_-IgA and IAPP_O_-IgA analyses were diluted 1:160 and 1:60, respectively, with blocking buffer. Fecal samples were diluted 1:18. Following incubation, plates were rinsed five times with PBST. Antibody binding was detected with horseradish peroxidase-conjugated polyclonal rabbit anti-human IgA (DakoCytomation, Glostrup, Denmark) diluted 1:2000 with blocking buffer and incubated for 1 h at RT with agitation. After three rinses with PBST, peroxidase substrate (SeraCare, Milford, MA, USA) was applied to each well, and the reaction was allowed to proceed until sufficient signal was developed. The reaction was terminated by the addition of 1M H_2_SO_4_. The end-point optical densities (OD) were read immediately at a wavelength of 450/540 nm on a microwell plate reader (EONTM, BioTek, Winooski, VT, USA). All samples had respective BSA controls where the wells were coated with 1 g/L BSA in PBS, and the procedure was followed as described above. Additionally, three inter-control samples in either plasma IAPP_M_-IgA or IAPP_O_-IgA, or fecal IAPP_O_-IgA analysis were applied to estimate the reproducibility of the signal throughout the analysis. The coefficients of variation for inter-controls were: 7% in plasma IAPP_M_-IgA analysis, 2% in plasma IAPP_O_-IgA analysis, and 15% in fecal IAPP_O_-IgA analysis. The autoantibody levels in plasma and feces were defined as OD (450–540 nm). Lastly, due to the high variability in fecal viscosity, the total protein content in the fecal samples was normalized before the IAPP_O_-IgA detection.

### 5.8. Detection of Total IgA in Plasma and Fecal Samples

Plasma and fecal total IgA levels were quantified using a Human IgA ELISA Kit (Mabtech, Nacka Strand, Sweden) according to the manufacturer’s instructions. Due to the high variability in fecal viscosity, the total protein content in fecal samples was normalized before the total IgA detection.

### 5.9. Detection of Albumin in Faecal Samples

Fecal albumin levels were determined using a Human Albumin ELISA Kit (Nordic BioSite, Täby, Sweden) according to the manufacturer’s instructions. Due to the high variability in fecal viscosity, the total protein content in fecal samples was normalized before the albumin detection.

### 5.10. Statistical Analyses

All statistical analyses were performed using SPSS software (version 29). The data were analyzed using either the Mann–Whitney U test (to assess plasma IAPP_O_-IgA levels between *APOE4* carriers and non-carriers at different time points), Kruskal–Wallis test (to assess the impact of T2D management strategy on plasma IAPP, IAPP_M_-IgA, or IAPP_O_-IgA levels at different time points), Wilcoxon matched-pairs signed rank test (to assess individual alterations in plasma IAPP, IAPP_M_-IgA, IAPP_O_-IgA, or total IgA levels post-diet in general and when the cohort was stratified upon patients with and without diabetic complications or *APOE4* status; to assess individual alterations in fecal IAPP, IAPP_O_-IgA, or total IgA levels post-diet in general), or Univariate Analysis of Variance (to assess the difference in plasma IAPP levels between different time points using age, gender, *APOE4* status, BMI, and status of diabetic complications as covariates). The correlation analyses between baseline values were performed using Spearman’s and partial correlation tests (two-tailed significance), the latter correcting for age, gender, *APOE4*, and BMI. The correlation analyses between the changes in values (month 3 minus baseline) were also performed using Spearman’s and partial correlation tests, the latter correcting only for BMI since age, gender, and *APOE4* were considered to be constant variables. The differences and correlations were considered significant at *p* ≤ 0.05. The power analysis has been described in a previous study [49]. In short, at most *n* = 9 patients were needed to demonstrate clinically important differences in weight, systolic blood pressure, total cholesterol, and HbA1c, and *n* = 18 patients in diastolic blood pressure with 80% power at 5% significance level. Hence, in total *n* = 30 patients were included to consider possible dropouts.

## 6. Conclusions

To summarize, following the O-BN diet for 3 months is sufficient to reduce levels of both IAPP and autoantibodies against IAPP in plasma, with the autoantibodies against IAPP oligomers persisting even after 4 months after returning to normal diets. Also, patients with more advanced diabetes, i.e., with diabetic complications as well as non-*APOE4* carriers, experience greater benefits from the diet. Nevertheless, following a healthier diet, such as the O-BN diet, might be of substantial importance to all T2D patients and should be encouraged to help in managing, delaying, or even preventing this devastating disease.

## Figures and Tables

**Table 1:** Clinical characteristics of the cohort at baseline.

Variables:	T2D (*n* = 30)
Age (mean years ± SD)	58 ± 8
Sex (M/F)	13/17
BMI (mean kg/m^2^ ± SD)	29.8 ± 4.2
APOE genotype, no (%)	
APOE23	5 (17%)
APOE24	3 (10%)
APOE33	14 (47%)
APOE34	8 (27%)
Diabetic complications, no (%)	19 (63%)
Retinopathy	10 (33%)
Microalbuminuria	5 (17%)
Peripheral neuropathy	4 (13%)
Macroangiopathy	5 (17%)
Autonomous neuropathy	7 (23%)
GI dysmotility	1 (3%)
T2D management, no (%)	
Diet alone	3 (10%)
Insulin	4 (13%)
Metformin	15 (50%)
Insulin + metformin	8 (27%)

APOE—Apolipoprotein E, BMI—body mass index, F—female, GI—gastrointestinal, M—male, T2D—Type 2 Diabetes.

**Table 2:** IAPP correlates with metabolic and peripheral inflammation markers in plasma.

	IAPP ^a^	
	Baseline (^c^/^d^)	Changes (^e^/^f^) ^b^
**Metabolic markers**		
BMI	0.465 */na	0.416 */na
Glucose	ns/ns	0.549 **/ns
C-peptide	ns/ns	0.449 */ns
Insulin	0.465 */ns	0.669 ***/ns
HOMA-IR	0.490 **/ns	0.704 ***/ns
GIP	ns/ns	0.407 */ns
Butyric acid	−0.460 */ns	ns/ns
Cholesterol	ns/0.419 *	ns/ns
LDL	ns/0.440 *	ns/ns
Albumin	ns/ns	ns/0.415 *
**Peripheral inflammation markers**		
Total IgA	ns/0.454 *	ns/ns
CRP	0.657 ***/ns	ns/ns
IFNγ	0.378 */0.784 ***	ns/ns
IL1α	ns/0.716 ***	ns/ns
IL1β	ns/0.516 *	0.398 */ns
IL2	ns/0.648 ***	0.416 */0.491 *
IL4	ns/0.648 ***	0.377 */ns
IL12p70	ns/0.633 ***	0.398 */ns
IL18	ns/0.569 **	ns/ns
TNFα	ns/0.726 ***	ns/ns
Resistin	ns/ns	−0.426 */ns
**Markers of liver damage**		
GGT	0.535 **/ns	ns/ns
**Markers of brain changes**		
NfL	ns/ns	−0.481 **/ns

^a^ *n* = 28, ^b^ indicates a change in values after 3 months compared to baseline, ^c^ before correcting for age, gender, *APOE4*, and body mass index (BMI), ^d^ after correcting for age, gender, *APOE4*, and BMI, ^e^ before correcting for BMI, ^f^ after correcting for BMI. CRP—C-reactive protein, GGT—Gamma-glutamyl transpeptidase, GIP—Glucose-dependent Insulinotropic Polypeptide, HOMA-IR—Homeostatic Model Assessment for Insulin Resistance, IAPP—Islet Amyloid Polypeptide, IFN—Interferon, IgA—Immunoglobulin A, IL—Interleukin, LDL—low-density lipoprotein, na—the correlation analysis is not applicable, NfL—Neurofilament light chain, ns—not significant, TNF—tumor necrosis factor. The data were analyzed with two-tailed Spearman’s (analyzing ^c^ and ^e^) and two-tailed partial (analyzing ^d^ and ^f^) correlation tests and presented as correlation coefficients. * Significant at *p* ≤ 0.05 level. ** Significant at *p* ≤ 0.01 level. *** Significant at *p* ≤ 0.001 level.

**Table 3:** IAPP_M_-IgA and IAPP_O_-IgA correlate with metabolic and peripheral inflammation markers in plasma.

	IAPP_M_-IgA		IAPP_O_-IgA	
	Baseline (^c^/^d^)	Changes (^e^/^f^) ^b^	Baseline (^c^/^d^)	Changes (^e^/^f^) ^b^
**Metabolic markers**				
IAPP ^a^	ns/0.431 *	0.394 */ns	ns/ns	ns/ns
Glucose	0.464 **/ns	ns/ns	0.455 */ns	ns/ns
HbA1c	0.383 */ns	ns/ns	ns/ns	ns/ns
Insulin	ns/ns	ns/ns	ns/ns	0.485 **/ns
HOMA-IR	ns/ns	ns/0.407 *	ns/ns	0.523 **/ns
Glucagon	ns/ns	0.387 */ns	0.429 */ns	0.389 */ns
GIP	ns/ns	0.565 ***/ns	0.365 */ns	ns/ns
Isobutyric acid	ns/ns	ns/ns	ns/ns	ns/0.806 ***
Triglycerides	ns/ns	0.559 ***/0.465 *	ns/ns	0.455 */ns
**Peripheral inflammation markers**				
Total IgA	0.779 ***/0.618 ***	ns/ns	0.530 **/ns	ns/ns
CRP	ns/ns	ns/ns	0.376 */ns	ns/ns
IL1α	ns/0.427 *	ns/ns	ns/ns	ns/ns
IL1β	ns/0.470 *	ns/ns	ns/ns	ns/ns
IL2	ns/0.481 *	ns/ns	ns/ns	ns/ns
IL18	ns/0.416 *	ns/ns	ns/ns	ns/ns
IFNγ	ns/0.392 *	ns/ns	ns/ns	ns/ns
TNFα	ns/0.453 *	ns/ns	ns/ns	ns/ns
**Markers of liver damage**				
GGT	0.418 */ns	ns/ns	0.550 **/ns	ns/ns
ALT	0.508 **/ns	ns/ns	ns/ns	ns/ns
**Markers of brain changes**				
NfL	ns/ns	ns/ns	ns/0.506 **	ns/ns

^a^ *n* = 28, ^b^ indicates a change in values after 3 months compared to baseline, ^c^ before correcting for age, gender, *APOE4*, and body mass index (BMI), ^d^ after correcting for age, gender, *APOE4*, and BMI, ^e^ before correcting for BMI, ^f^ after correcting for BMI. ALT—Alanine aminotransferase, CRP—C-reactive protein, GGT—Gamma-glutamyl transpeptidase, GIP—Glucose-dependent Insulinotropic Polypeptide, HbA1—Hemoglobin A1C, HOMA-I—Homeostatic Model Assessment for Insulin Resistance, IAPP—Islet Amyloid Polypeptide, IgA—Immunoglobulin A, M—Monomer, ns—not significant, O—Oligomer. The data were analyzed with two-tailed Spearman’s (analyzing ^c^ and ^e^) and two-tailed partial (analyzing ^d^ and ^f^) correlation tests and are presented as correlation coefficients. * Significant at *p* ≤ 0.05 level. ** Significant at *p* ≤ 0.01 level. *** Significant at *p* ≤ 0.001 level.

**Table 4:** Nutrition composition and daily mean intake of energy of the modified Okinawa-based Nordic diet compared with the Nordic Nutrition Recommendations (NNR) 2012.

Nutritional Value	Unit	Calculated Value	E%	Recommended (NNR 2012)
Total Energy	kcal	1866.0		
Energy (excluding beverages)	kcal	1629.0		
Carbohydrates	g	168.4	42 E%	45–60 E%
Sucrose	g	23.5	6 E%	<10 E%
Dietary fiber	g	35.9	4 E%	25–35 g
Fat	g	63.9	35 E%	25–40 E%
Saturated fatty acids	g	18.7	10 E%	<10 E%
Polyunsaturated fatty acids	g	14.9	8 E%	5–10 E%
Monounsaturated fatty acids	g	17.8	10 E%	10–20 E%
Protein	g	95.0	23 E%	10–20 E%
Alpha-Tocopherol	mg	1.9		
Beta-Carotene	µg	9902.1		
Retinol	µg	259.7		
Vitamin A	µg	139.9		700
Vitamin D	µg	8.8		10
Vitamin E	mg	11.4		8
Thiamine	mg	1.1		1.1
Riboflavin	mg	1.2		1.2
Niacin equivalent	mg	34.5		14
Niacin	mg	19.7		14
Vitamin B6	mg	2.1		1.2
Folate	µg	386.1		300
Vitamin B12	µg	10.4		2
Vitamin C	mg	303.0		75
Sodium	mg	2401.1		2300
Potassium	mg	3385.6		3100
Phosphorous	mg	1446.7		600
Calcium	mg	840.4		800
Iron	mg	10.7		15
Magnesium	mg	317.4		280
Zinc	mg	9.7		7
Iodine	µg	34.9		150
Selenium	µg	61.0		50

## Data Availability

The data presented in this study are available on reasonable request from the corresponding author.

## Supplementary Materials

The following supporting information can be downloaded at: https://www.mdpi.com/article/10.3390/ijms25147665/s1.
