# Supplementary material for: Okinawa-Based Nordic Diet Decreases Plasma Levels of IAPP and IgA against IAPP Oligomers in Type 2 Diabetes Patients
Source: Int J Mol Sci. 2024 Jul 12;25(14):7665. doi: 10.3390/ijms25147665 (PMC11276895; doi:10.3390/ijms25147665)
Supplement: Supplementary file 1 [file ijms-25-07665-s001.zip › ijms-3075808-supplementary.pdf]

Supplementary material

Supplementary Table S1. Breakfast composition in patients at baseline, during the 3 months-long dietary intervention, and after the intervention.

	**Breakfast at baseline**					**Breakfast during and after the intervention**				
**no.**	**Energy (kcal)**	**Protein (g/E%)**	**Fat (g/E%)**	**Carb (g/E%)**	**Fiber (g/E%)**	**Energy (kcal)**	**Protein (g/E%)**	**Fat (g/E%)**	**Carb (g/E%)**	**Fiber (g/E%)**
1	488	22/18	33/59	25/20	6/2	331	17/21	17/46	24/30	7/4
2	184	7/15	3/15	29/64	6/7	258	13/20	10/33	27/42	7/5
3	301	12/16	22/63	14/19	3/2	267	16/24	15/50	15/23	4/3
4	191	10/20	12/55	11/24	0.7/0.7	420	28/28	16/34	37/36	5/2
5	209	8/16	5/21	31/60	5/5	248	10/17	8/28	30/49	8/6
6	241	8/14	5/17	38/64	8/6	81	3/15	2/26	10/52	3/8
7	515	20/16	21/36	55/43	12/5	362	24/27	13/33	33/37	5/3
8	230	10/18	11/40	22/39	3/2	537	33/25	19/32	53/40	8/3
9	526	26/20	24/40	49/38	5/2	309	23/30	10/29	31/41	5/3
10	260	9/14	12/41	28/43	3/2	248	14/23	9/30	26/42	5/5
11	119	7/24	6/41	9/30	3/5	131	8/25	7/44	8/26	3/5
12	298	10/13	9/26	43/58	5/3					
13	328	16/19	3/9	56/70	5/3	310	13/17	10/29	39/51	5/3
14	443	18/16	20/41	45/41	5/2	141	8/22	6/40	12/35	3/3
15	332	11/14	8/22	51/62	4/2	221	16/30	5/21	25/46	4/4
16	213	7/13	3/13	36/69	5/5	253	15/24	9/32	24/39	6/5
17	291	10/14	14/43	28/40	8/5	537	15/12	33/55	40/31	9/3
18	497	21/17	27/48	41/34	4/1	188	11/24	4/19	14/30	4/4
19	300	12/16	13/38	31/43	10/6	385	18/19	15/35	42/44	8/4
20	343	13/16	16/40	33/40	8/4	175	9/21	9/44	14/32	4/4
21	383	15/16	11/26	51/54	8/4	299	10/13	6/17	49/67	8/5
22	590	25/17	29/44	55/38	4/1					
23	390	17/17	11/26	51/53	8.5/4	364	14/16	15/37	38/42	9/5
24	555	19/14	26/42	56/41	11/4	488	25/21	15/27	58/48	9/3
25	502	25/20	18/32	57/46	5/2	341	19/23	17/43	27/32	5/3
26	212	10/19	4/17	31/59	6/5	265	15/23	3/11	39/60	9/6
27	465	20/18	18/32	53/46	4/2	355	24/27	14/35	31/36	5/3
28	290	15/21	17/53	18/25	2/1					
29	153	6/17	3/19	24/65	4/4	305	15/20	9/27	38/51	8/5
30	454	21/19	16/31	52/47	7/3	374	20/21	11/26	45/50	7/3

n=30. Carb - carbohydrates, E% - energy percentage, no. – patient number.

Supplementary Table S2. Description of food items included in the Okinawa-based Nordic diet.

**Food groups**	
Root vegetables	Hot or cold. Red or yellow carrots as snacks or incorporated in the dish. Parsnips, parsley root, rutabaga, celery root, beetroot, artichoke, or sweet potatoes as part of the dishes.
Potatoes	Shredded in a potatoe burger, fried.
Vegetables	Onion, leek, garlic, cabbage, cauliflower, broccoli, squash, eggplant, fennel, spinach, various types of salad, tomato, pepper, cucumber, mushroom, and asparagus.
Legumes	Fresh; green peas, sweet peas, and soybean (edamame).<br>Dried; white and black beans, kidney beans, and lentils.
Nuts	Walnuts, almonds, and cashews.

Seeds	Sunflower seeds, pumpkin seeds, linseed, and sesame seeds, as part of snacks served with fruits and berries.
Fruits and berries	Apple, orange, pear, strawberry, blueberry, lingonberry, dried apricots, and prunes. Coconut milk was used for cooking.
Meat products and poultry	Chicken, rooster, turkey, game meat (hart and deer), and ground beef.
Fish and seafood	Salmon, codfish, plaice, mackerel, herring, and prawns.
Eggs	Eggs were used for cooking and as snacks.
Dairy products	Low-fat drinking milk (maximum 1.5% fat), filmjölk (a Swedish fermented product similar to yogurt) and low-fat yogurt (1.5% fat), hard cheese (17% fat), parmesan cheese, cottage cheese, and quark. Whipped cream (36% fat) and soy cream used for cooking, and Turkish yogurt (10% fat).
Fat and oil	Vegetable oil including rapeseed oil, sesame oil, and olive oil for cooking and dressing. Vegetable fat spread (60% fat) for bread.
Rice	Whole-grain rice and black Thai rice. A maximum of half a dl of uncooked rice per serving.
Cereals	Rolled oats, rye flakes, whole-grain whole-kernel rye, oat, barley, and wheat. Bread rich in whole-grain, a maximum of two slices per day. Whole-grain pasta, bean paste, millet, oat bran, quinoa, and rye flour.
Beverages/liquids (non-alcoholic)	Recommended free amount of tap water or mineral water with the meals. Light products to be avoided. Tea, filtered coffee, or instant coffee.
Spices	Own preference with restrictions on salt.
Sweets and desserts	Homemade dark chocolate (>70%), bean truffles, coconut flakes, ginger, and prune cake. No refined sugar or sweetener was used, but instead natural sweet foods like prunes, pears, and occasionally honey.
Alcoholic beverages	Maximal intake of alcoholic beverages was set to 30 g ethanol/week.

Supplementary Table S3. Concentrations of metabolic, hormonal, and inflammatory markers as well as brain-related changes after an intervention with the Okinawa-based Nordic diet.

		95% CI		
	**Mean**	**Lower**	**Upper**	**p-value**
Acetic acid (µM)				
Baseline	302.8	256.0	349.6	
3rd month	283.5	226.0	341.0	0.452
7th month	328.4	272.2	384.6	0.302
Albumin (g/l)				
Baseline	39.1	38.0	40.2	0.560
3rd month	38.8	37.6	40.0	0.523
7th month	39.4	38.2	40.7	
ALT (µkat/l)				
Baseline	0.53	0.12	1.30	
3rd month	0.42	0.03	1.50	<0.001
7th month	0.44	0.12	1.40	0.005
Butyric acid (µM)				
Baseline	14.1	13.0	15.2	
3rd month	15.4	12.7	18.1	0.370
7th month	11.8	10.5	13.0	0.007
C-peptide (nM)				
Baseline	0.99	0.82	1.2	
3rd month	0.88	0.70	1.0	0.022
7th month	0.88	0.68	1.1	0.131
Calprotectin (faeces; mg/kg)				

Baseline	141.1	71.8	210.5	
3rd month	137.7	60.8	214.5	0.878
7th month	131.1	37.0	225.2	0.778
Calprotectin (serum; ng/ml)				
Baseline	1816	1502	2130	
3rd month	2187	1785	2588	0.074
7th month	1460	1003	1916	0.129
CCK (pg/ml)				
Baseline	27	21	32	
3rd month	20	14	26	0.005
7th month	23	16	30	0.267
Cholesterol (mM)				
Baseline	4.65	4.36	4.95	
3rd month	4.22	3.87	4.57	0.001
7th month	4.71	4.37	5.05	0.636
CRP (mg/l)				
Baseline	3.7	1.8	5.6	
3rd month	2.9	1.0	4.8	0.569
7th month	1.7	-0.5	3.9	0.173
Glucose (mM)				
Baseline	9.71	8.54	10.9	
3rd month	7.91	6.55	9.27	<0.001
7th month	9.28	7.71	10.8	0.466
GGT (μkat/l)				
Baseline	0.80	0.53	1.07	
3rd month	0.58	0.30	0.85	0.016
7th month	0.53	0.17	0.89	0.073
Ghrelin (pg/ml)				
Baseline	852	608	1094	
3rd month	798	555	1041	0.219
7th month	728	481	975	0.012
GIP (pg/ml)				
Baseline	78	57	99	
3rd month	55	34	76	<0.001
7th month	75	52	98	0.692
GLP-1 (pg/ml)				
Baseline	2.7	1.8	3.5	
3rd month	1.8	0.9	2.7	0.013
7th month	2.4	1.4	3.3	0.477
Glucagon (pg/ml)				
Baseline	1.9	1.3	2.6	
3rd month	1.3	0.6	1.9	0.003
7th month	1.7	1.1	2.4	0.426
Haptoglobin (g/l)				
Baseline	1.4	1.3	1.6	
3rd month	1.5	1.3	1.7	0.605
7th month	1.3	1.2	1.5	0.009
HbA1c (mM)				
Baseline	61.6	56.4	66.7	
3rd month	49.2	44.0	54.4	<0.001
7th month	54.4	48.8	59.9	0.002
HDL (mM)				
Baseline	1.22	1.10	1.35	
3rd month	1.19	1.05	1.32	0.267
7th month	1.34	1.20	1.47	0.003
IFNγ (pg/ml)				
Baseline	778	-	-	

3rd month	643	-	-	0.12
7th month	656	-	-	0.35
IL1α (pg/ml)				
Baseline	6.6	-	-	
3rd month	5.6	-	-	0.18
7th month	6.0	-	-	0.56
IL1β (pg/ml)				
Baseline	12.1	-	-	
3rd month	10.3	-	-	0.17
7th month	10.8	-	-	0.49
IL12p70 (pg/ml)				
Baseline	490	-	-	
3rd month	415	-	-	0.07
7th month	458	-	-	0.61
IL18 (pg/ml)				
Baseline	226	-	-	
3rd month	189	-	-	0.001
7th month	201	-	-	0.09
IL2 (pg/ml)				
Baseline	315	-	-	
3rd month	321	-	-	0.86
7th month	357	-	-	0.41
IL4 (pg/ml)				
Baseline	98.1	-	-	
3rd month	79.9	-	-	0.17
7th month	89.6	-	-	0.66
Insulin (mIU/l)				
Baseline	16	13	18	
3rd month	12	9	15	0.004
7th month	13	10	16	0.089
Isobutyric acid (μM)				
Baseline	21.0	19.8	22.2	
3rd month	19.8	18.5	21.2	0.129
7th month	19.7	18.1	21.3	0.139
Isovaleric acid (μM)				
Baseline	63.0	58.6	67.5	
3rd month	57.3	52.9	61.8	0.018
7th month	62.0	56.9	67.1	0.707
LDL (mM)				
Baseline	2.92	2.62	3.22	
3rd month	2.68	2.33	3.03	0.041
7th month	2.82	2.49	3.16	0.356
Leptin (pg/ml)				
Baseline	9404	587	1294	
3rd month	5850	199	971	<0.001
7th month	8553	491	1219	0.171
NfL (pg/ml)				
Baseline	21.3	-	-	
3rd month	26.7	-	-	0.049
7th month	23.1	-	-	0.18
PAI-I (ng/ml)				
Baseline	72.4	58.9	86.0	
3rd month	66.4	52.4	80.4	0.082
7th month	71.8	56.7	86.9	0.889
PYY (ng/ml)				
Baseline	1.9	1.8	2.1	
3rd month	1.5	1.4	1.7	<0.001

7th month	1.7	1.5	1.8	0.002
Propionic acid (μM)				
Baseline	17.6	16.3	18.8	
3rd month	18.2	15.4	21.0	0.657
7th month	16.7	15.2	18.2	0.253
Resistin (pg/ml)				
Baseline	5234	3904	6565	
3rd month	5043	3705	6382	0.683
7th month	4325	2915	5736	0.082
TNFα (pg/ml)				
Baseline	14.9	-	-	
3rd month	12.5	-	-	0.14
7th month	13.6	-	-	0.60
Triglycerides (nM)				
Baseline	1.79	1.41	2.16	
3rd month	1.49	1.09	1.89	0.009
7th month	1.96	1.46	2.46	0.367
Visfatin (pg/ml)				
Baseline	998	732	1264	
3rd month	1115	816	1415	0.235
7th month	812	535	1089	0.021
Zonulin (faeces; ng/ml)				
Baseline	49.4	38.3	60.4	
3rd month	92.3	71.3	113.2	<0.001
7th month	53.5	40.4	66.6	0.572
Psych. well-being (score)				
Baseline	22.6	0.0	84.0	
3rd month	12.2	0.0	48.0	0.003
7th month	12.0	0.0	53.0	0.050

Values are presented as absolute mean values and 95% confidence interval (CI) with lower and upper limits at inclusion (baseline) (n=30), 3 months after the dietary intervention (n=30), and 4 months after the end of the dietary intervention (7th month; n=23). ALT – alanine aminotransferase, CCK – cholecystokinin, CRP – C-reactive protein, GGT – gamma-glutamyl transpeptidase, GIP – glucose-dependent insulinotropic polypeptide, GLP-1 – glucagon-like peptide-1, HbA1c – hemoglobin A1c, HDL – high-density lipoprotein, IFNγ - interferon γ, IL – interleukin, LDL – low-density lipoprotein, NfL – neurofilament light chain, PAI-I – plasminogen activator inhibitor-1, PYY – polypeptide YY, TNFα - tumor necrosis factor α. Comparisons were made between baseline and 3rd month and 7th month. P≤0.05 indicates a statistically significant difference.

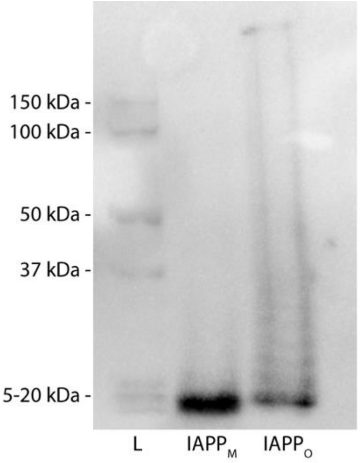

Supplementary Figure S1. Characterization of IAPP self-assembly products. Western-Blot analysis of IAPP monomers ($IAPP_M$) and oligomers ($IAPP_O$). L – ladder (#1610377, BioRad, Sweden).
